# Multierosive Juvenile Idiopathic Arthritis With Positive Rheumatoid Factor: A Case Report of a Young Girl Followed at the University Clinics of Bukavu

**DOI:** 10.1155/crrh/9884531

**Published:** 2026-07-26

**Authors:** Delphin Murhula Katabana, Théophile Barhwamire Kabesha, Rodrigue Mupenda Mwenibamba, Roland Lwandiko Cibenda, Paul Ngongo Tshonda, Archippe Birindwa Muhandule, Philippe Katchunga Bianga, Tony Shindano Akilimali

**Affiliations:** ^1^ Internal Medicine Department, Bukavu University Clinics, Official University of Bukavu, Bukavu, Democratic Republic of the Congo; ^2^ Ophthalmology Department, Bukavu University Clinics, Official University of Bukavu, Bukavu, Democratic Republic of the Congo; ^3^ Orthopedic Surgery Department, Bukavu University Clinics, Official University of Bukavu, Bukavu, Democratic Republic of the Congo; ^4^ Pediatric Department, Bukavu University Clinics, Official University of Bukavu, Bukavu, Democratic Republic of the Congo

**Keywords:** diagnostic delay, DR Congo, juvenile idiopathic arthritis, multierosive, rheumatoid factor

## Abstract

Polyarticular juvenile idiopathic arthritis (JIA) with positive rheumatoid factor is a rare and severe form of chronic inflammatory rheumatism in children. We report the case of a young girl treated at the University Clinics of Bukavu (eastern DR Congo), whose disease began at the age of 10. The course of the disease was characterized by highly inflammatory polyarthritis with severe multierosive lesions. Immunological testing revealed positivity for rheumatoid factor and anti‐CCP antibodies. Diagnosis and treatment were delayed for approximately nine years (2008–2017). Management was complicated by poor adherence and discontinuation of methotrexate and corticosteroids. The patient subsequently died to recurrent infectious complications and hypertension. This case illustrates the impact of diagnostic delay and difficulties in accessing care on the prognosis of severe forms of JIA in resource‐limited settings.

## 1. Introduction

Juvenile idiopathic arthritis (JIA) is a group of chronic arthritis conditions of unknown cause that occur before the age of 16 and last for more than six weeks [1]. The polyarticular form, affecting at least five joints in the first six months, includes two entities: rheumatoid factor (RF)‐negative JIA (15%–20%), sometimes associated with uveitis, with a peak incidence between 1–3 years and 9–14 years, and RF‐positive JIA (RF + JIA), which accounts for about 5% of cases and is like rheumatoid arthritis (RA) in adults. The last one, an autoimmune disease that mainly affects girls aged 10–12, has a high potential to lead to disability [2, 3].

The prevalence and incidence of JIA vary depending on the study, population, and classification system (ACR, EULAR, ILAR) [4]. The global prevalence is estimated to be between 3.8 and 400 per 100,000 children. For JIA RF+, the incidence is 0.4 (0.3–0.5) and the prevalence is 1.0 (0.7–1.3) per 100,000 children, with a sex ratio of 7–9 girls for one boy [5–7].

Clinically, RF + JIA is like RA in adults, with bilateral and symmetrical involvement, predominantly affecting the small joints of the hands (wrists and fingers), feet (ankles and metatarsophalangeal joints), and large joints (knees and hips). The cervical spine and temporomandibular joints (TMJ) are often affected, unlike the thoracolumbar spine and sacroiliac joints [8, 9]. Moderate inflammatory response is observed in two‐thirds of cases, with positive RF and anti‐CCP antibodies [10].

The early onset of this disease is often responsible for delayed diagnosis, noncompliance with treatment, and progression to severe joint erosion in adulthood in the absence of adequate management [11, 12]. The progression may be marked by inflammatory flare‐ups or a continuous process, leading to capsuloligamentous retraction or osteocartilaginous destruction. Functional disability is noted in 20%–30% of cases, often associated with growth retardation [11, 13].

Treatment is multidisciplinary, including pediatrician, pediatric rheumatologist, ophthalmologist, physical therapist, occupational therapist, pediatric orthopedist, and psychologist as needed [8, 12, 14]. Initial treatment is based on nonsteroidal anti‐inflammatory drugs (NSAIDs) and early functional rehabilitation. If this fails, intra‐articular injections of slow‐release corticosteroids are indicated. Long‐term treatment combines methotrexate (first‐line drug) and biologics in cases of recurrent or severe forms. The introduction of these biologics has led to a significant improvement in the overall prognosis [14, 15]. However, remissions in RF + JIA remain rare (10%) and require prolonged therapy to limit joint destruction. In addition, biological treatments expose patients to an increased risk of infection; hence, the importance of adhering to the vaccination schedule, particularly against influenza and pneumococcus, excluding live attenuated vaccines [8, 14, 15].

In countries with limited resources, late diagnosis, often at a stage of severe functional disability, complicates management. Extra‐articular complications (cardiovascular, pulmonary, renal, and infectious) worsen the prognosis and socioeconomic burden.

The DRC has a three‐tiered healthcare system (peripheral, intermediate, and central), but access to care remains limited by poor infrastructure, insecurity, and low public funding [16]. In 2014, 42% of healthcare expenditures were borne directly by households, and the situation has deteriorated since then [17, 18]. Diagnostic capabilities are insufficient, with a lack of equipment and specialists (particularly in pediatric rheumatology) delaying the diagnosis of JIA [19]. Treatment relies primarily on anti‐inflammatory drugs, corticosteroids, and methotrexate when available. As in many African countries, biological therapies remain inaccessible to most patients, contributing to a poorer functional prognosis [20].

The underdiagnosis of JIA, like other autoimmune diseases in these contexts, justifies the presentation of this case followed for 5 years, highlighting the importance of early diagnosis and management.

## 2. Patient Information

We report the case of a 22‐year‐old female patient with no significant past medical or family history, referred by an orthopedist in February 2020 for persistent distal polyarticular pain. The clinical history reveals that symptoms began at the age of 10, characterized by painful joint swelling in the hands and toes, as well as limping. Initial self‐medication with indigenous products, followed by treatments for acute rheumatic fever, led to temporary remissions. The parents then noticed stunted growth and loss of manual function. In 2017, tests revealed a positive RF (qualitative test), bone erosions, tendon retractions, and severe joint damage including the ankles.

Irregular use of NSAIDs and low‐dose methotrexate has been reported. In 2020, an inflammatory flare‐up prompted a referral to internal medicine for reassessment.

Timeline.

⁃ 2008: Onset of the disease at age 10. Symmetrical inflammatory involvement of the joints in the hands and feet. The exact number of affected joints and results of laboratory tests were not specified.
⁃ 2010: Deformities of the knees and left elbow.
⁃ 2014: Surgical arthrolysis of the left elbow and rehabilitation.
⁃ 2017: Suspected RA. The RF was positive qualitatively. X‐rays showed erosion and ankylosis. The ophthalmological examination revealed ametropia without uveitis. Treatments included NSAIDs, analgesics, oral corticosteroid therapy, intra‐articular corticosteroid injections, and antibiotics for infections. Initiation of methotrexate treatment without adherence, followed by discontinuation after 1 year.
⁃ 2019: Acupuncture, temporary improvement, ankylosis of the knees.
⁃ 2019–2020: New inflammatory flare‐ups, hospitalizations for infections.
⁃ 2021: Mobilization of the right knee under general anesthesia and wearing of a Zimmer‐type reinforced knee brace.
⁃ 2022: Arthrodesis of the right talocrural joint, inflammatory recurrences.
⁃ 2023–2024: Discovery of high blood pressure, bilateral edema of the lower limbs; hospitalization then loss of follow‐up.

**FIGURE 1:**
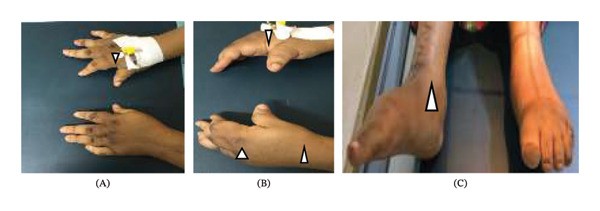
Multiple deformities of the fingers, buttonhole deformity, swan neck deformity with Z‐shaped thumb, misalignments, and retractions, and ankylosis of the wrists and ankles (A), (B), and (C).

Clinical findings:

In July 2022, clinical examination showed a patient in a wheelchair, altered general condition, WHO = 3, HAQ = 3, BP = 145/95 mmHg, pulse = 85 bpm, temperature = 36.5°C. Rheumatological examination noted: micrognathia, DAS28 = 4.7, 4 swollen joints (knees and elbows), and 10 painful joints (IPP and MCP) with VAS = 6/10. Typical deformities: ulnar windlass deformity, buttonhole fingers, swan neck deformity, Z‐shaped thumb, and digital misalignments (Figure 1A,B). Lower limbs: limitation and deviation of the right knee, ankle stiffness, and pitting edema. Cardio‐respiratory, abdominal, dermatological, and ophthalmological examinations were normal.

### 2.1. Diagnostic Assessment

Paraclinical tests performed: Complete blood count: hemoglobin (Hb) = 10 g/dL, hematocrit (Htc) = 30%, mean corpuscular volume (MCV) = 70.6 fL, white blood cells (WBC) = 8.3 G/L, platelets = 182 G/l. C‐reactive protein (CRP) = 10 mg/dL. Normal creatinine = 0.6 mg/dL, negative proteinuria, normal ionogram and blood sugar levels. Liver function tests: normal transaminases. Serology: Human Immunodeficiency Virus, Hepatitis B Virus, Hepatitis C Virus, and syphilis are all negative. Immunology: Antistreptolysin O (ASLO) = 20 IU/L, RF = 25 IU/mL, anticyclic citrullinated peptide antibodies (anti‐CCP) > 340 EU/mL, antinuclear antibodies (ANA) negative.

**FIGURE 2:**
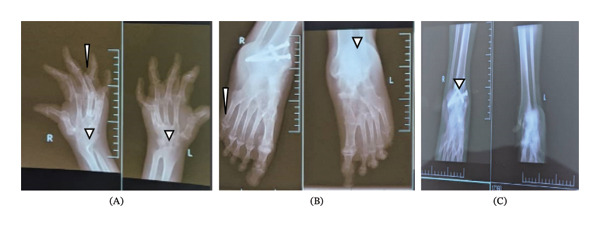
X‐rays of the hands and feet showing fused carpals and tarsals, MCP misalignments, PIP, MTP erosions, osteosynthesis materials after arthrodesis of the right talocrural joint (A), (B), (C).

**FIGURE 3:**
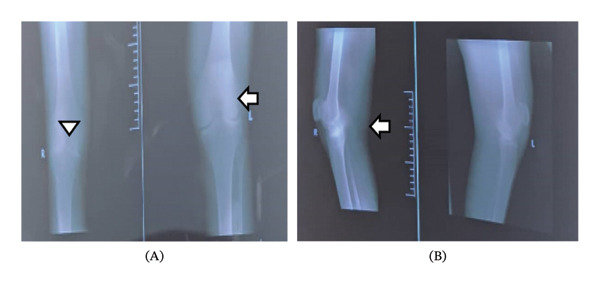
Front (A) and side (B) X‐rays of the knees showing ankylosis of the right knee with slight flexion deformity and left valgus.

Imaging: X‐rays of the hands and feet = advanced osteoarticular lesions (Figures 2 and 3). Cardiac assessment: normal pro‐B‐type natriuretic peptide (Pro‐BNP). Cardiovascular evaluation was unremarkable, with normal ProBNP levels, ECG( sinus rhythm, 86 beats per minute, QTc=420 ms, Cornell=8mm, Sokolov=16mm), echocardiography, and lower‐limb venous doppler ultrasoud.

Considering the findings, we concluded that the patient had a moderate flare‐up of advanced juvenile RA, multifusion, in the sequelae stage, complicated by high blood pressure. No other etiology of this hypertension was identified.

### 2.2. Therapeutic Intervention

The patient received anti‐inflammatory treatment with ketoprofen 100 mg, taken orally twice daily, and later as suppositories due to stomach pain, followed by oral corticosteroid therapy (prednisone 40 mg/day). After ruling out infection, we reintroduced methotrexate 15 mg/week per os, with a gradual reduction in corticosteroid therapy to less than 10 mg/day 3 months later. Treatment was continued for 3 years. Other treatments: esomeprazole, potassium, calcium‐vitamin D, folic acid, iron, and an antihypertensive agent (telmisartan + hydrochlorothiazide).

### 2.3. Follow‐Up and Outcome

The outcome was marked by clinical improvement with partial remission of pain despite joint sequelae. Multidisciplinary follow‐up was beneficial, with partial recovery of walking ability. Infectious episodes required antibiotic treatment. Subsequently, the patient developed depression and insomnia and was then lost to follow‐up. She died at home in an unspecified clinical condition.

## 3. Discussion

### 3.1. Epidemiology

RF‐positive JIA (RF + JIA) is the pediatric equivalent of RA in adults. This form of chronic inflammatory rheumatism remains underdiagnosed in sub‐Saharan Africa due to a lack of information and limited access to specialized care. In Cotonou (Benin), a study of 185 children with osteoarticular disorders found 34 cases of JIA (18.5%), of which 23% were RF‐positive JIA [21]. In Abidjan, the hospital prevalence of JIA in adult rheumatology was 0.36%, with 16.13% of cases being RF+. The average time to consultation was 13.5 months. In South Africa, higher rates were observed in black children, but overall, data remains scarce [22]. In the Democratic Republic of the Congo (DRC), no epidemiological data are currently available on JIA or on the prevalence of chronic inflammatory arthritis.

### 3.2. Clinical Description

RF + JIA manifests as bilateral, symmetrical polyarthritis, preferentially affecting the wrists, small joints of the fingers, ankles, metatarsophalangeal joints, knees, hips, cervical spine, and TMJs. These lesions cause stiffness, rigidity, and muscle‐tendon retractions. Extra‐articular signs are rare in this form [8, 9]. The clinical presentation of our patient is consistent with the classic manifestations of RF + JIA.

### 3.3. Laboratory Findings

In this patient, the inflammatory syndrome was moderate outside of infectious episodes. Inflammatory anemia persisted, with a slight elevation in CRP, without hyperleukocytosis. RF was positive, and anti‐CCP antibodies were significantly elevated, indicating an increased risk of joint destruction [10]. However, the biological diagnosis was made late: RF was only positive in 2017, and anti‐CCP was detected in 2022, more than 10 years after the first symptoms appeared. This delay was partly due to the limited availability of specialized tests. For example, in Bukavu, anti‐CCP testing requires outsourcing outside the country.

### 3.4. Medical Imaging

The radiological damage caused by RF + JIA is often severe and visible at an advanced stage: joint destruction of the wrists (carpal fusion), ankles (tarsal fusion), small joints of the fingers, metatarsophalangeal joints, hips, knees, cervical spine (ankylosis, atlantoaxial dislocation), and TMJs (with mandibular growth abnormalities) [11, 13]. In our patient, advanced joint damage and postoperative images of reconstructive surgery were observed.

### 3.5. Treatment

The treatment of RF + JIA is based on a multidisciplinary approach. Methotrexate is the first‐line treatment, combined with NSAIDs or corticosteroids. If this fails, biotherapies are introduced: anti‐TNF (etanercept, adalimumab), anti‐IL6 (tocilizumab), T‐cell costimulation inhibitors (abatacept), and more recently, JAK inhibitors (tofacitinib) [14, 15].

In our context, the lack of specialized resources for autoimmune diseases and the unavailability of biotherapies contribute to the worsening of joint damage. Hence the importance of setting clear therapeutic goals at the time of diagnosis and adapting treatments to control inflammation, prevent complications, and limit toxicity [14]. Reconstructive surgery may become necessary, as in the case of our patient, and comprehensive care including psychological support for the child and their family is essential.

### 3.6. Progression and Prognosis

Our patient illustrates the unfavorable progression of RF + JIA: 14 years after the onset of symptoms, she presented with persistent disease activity (DAS 28 = 4.7), severe disability (HAQ = 3), with a diagnostic delay of nearly 10 years and noncompliance with treatment. The literature indicates that 70% of RF + polyarticular JIA remains progressive in the long term, with erosion and functional disability in 20%–30% of cases, and often aggravating growth and weight retardation [8, 23]. Factors associated with poor prognosis include RF positivity, female gender, and persistent joint activity at 5 years [3]. Mortality, although low, is increased in patients with a history of JIA, particularly in polyarticular or systemic forms, which are often associated with other autoimmune diseases [8].

### 3.7. Patient Perspective

In our case, after a short remission, the patient expressed regret and said that the illness had ruined her childhood and youth. She died at home, possibly because of an infection or cardiovascular complication.

## 4. Conclusion

RF + JIA, a juvenile form of RA, is a rare but severe disease that mainly affects young girls. Its chronic progression with repeated inflammatory flare‐ups causes irreversible joint damage and major disability in adulthood. Early diagnosis, access to disease‐modifying treatments, and multidisciplinary follow‐up are essential to improve the prognosis. This clinical case illustrates the major challenges encountered in resource‐limited countries such as the DRC.

## Funding

The authors have nothing to report.

## Ethics Statement

The publication of this case report was approved by the Ethics Committee of the Official University of Bukavu.

## Consent

Written informed consent was obtained from the guardian of the deceased patient for the publication of this case report.

## Conflicts of Interest

The authors declare no conflicts of interest.

## Supporting Information

Additional supporting information can be found online in the Supporting Information section.

## Supporting information


**Supporting Information** CARE Checklist.

## Data Availability

The data that support the findings of this study are available on request. The data are not publicly available due to privacy or ethical restrictions.
